# Entamoeba Histolytica: Updates in Clinical Manifestation, Pathogenesis, and Vaccine Development

**DOI:** 10.1155/2018/4601420

**Published:** 2018-12-02

**Authors:** Micaella Kantor, Anarella Abrantes, Andrea Estevez, Alan Schiller, Jose Torrent, Jose Gascon, Robert Hernandez, Christopher Ochner

**Affiliations:** ^1^Kendall Regional Medical Center, Miami, FL, USA; ^2^Hospital Corporation of America, East Florida Division, Fort Lauderdale, FL, USA

## Abstract

*Entamoeba histolytica *is the responsible parasite of amoebiasis and remains one of the top three parasitic causes of mortality worldwide. With increased travel and emigration to developed countries, infection is becoming more common in nonendemic areas. Although the majority of individuals infected with* E. histolytica* remain asymptomatic, some present with amoebic colitis and disseminated disease. As more is learned about its pathogenesis and the host's immune response, the potential for developing a vaccine holds promise. This narrative review outlines the current knowledge regarding* E. histolytica* and* E. dispar* and insight in the development of a vaccine.

## 1. Introduction

Amoebiasis, or amoebic dysentery, is a term used to describe an infection caused by the protozoan* Entamoeba histolytica* [1]. Most infections are asymptomatic, but invasive intestinal disease may occur manifesting with several weeks of cramping, abdominal pain, watery or bloody diarrhea, and weight loss [1]. Disseminated, extra intestinal disease such as liver abscess, pneumonia, purulent pericarditis, and even cerebral amoebiasis has been described [1–3]. Worldwide, it has been estimated that up to 50 million people are affected by* E. histolytica*, primarily in developing countries, and it is responsible for over 100,000 deaths a year [4, 5]. Transmission generally occurs by the ingestion of infected water or food due to fecal excretion of cysts, and even fecal-oral transmission within household and during male homosexual activity [1, 2, 6]. In this review we will synthesize the current literature on clinical manifestations, pathogenesis, vaccine development, and the controversy regarding the pathogenic potential of* E. dispar.*

## 2. Methods

### 2.1. Data Sources and Searches

We searched Ovid MEDLINE/PubMed databases to identify relevant articles indexed from 1991 to 2018. The search was conducted between February 3, 2018 and April 6, 2018, looking for publications that contain “amoebiasis,” “Entamoeba histolytica,” “amoebic colitis,” and “extraintestinal amoebiasis” in the title or abstract. Additional relevant articles from the reference lists of relevant studies were also used.

### 2.2. Study Selection

Eligible articles were published in English. Case reports and case series were included. Given the relatively small number of studies published in this area, no further exclusion criteria were imposed.

## 3. Results and Discussion

### 3.1. Epidemiology

Amoebiasis is a worldwide problem; however, individuals living in developing countries are at greatest risk given poor sanitation and socioeconomic conditions. Due to the increase in emigration and travel from endemic areas, infection is becoming more common in developed countries such as those in North America. Areas with highest rate of infection include India, Africa, and Central and South America, particularly Mexico [7].

The rate of infection in amoebic colitis is almost identical between males and females [7, 8]. Amoebic liver abscess (ALA) is ten times more common in males than in females and commonly affects those between 18 and 50 years of age [1, 8, 9]. The reason for this disparity is unclear; however, it has been suggested that increased alcohol consumption leading to hepatocellular damage may be a predisposing factor in male patients [7, 10, 11]. It has also been posited that the relative iron deficiency or hormonal factors in women of child bearing age may be a protective factor against disseminated disease [11].

The Monthly Infectious Diseases Surveillance Report produced by the Public Health Ontario (PHO; [12]) estimated the annual incidence rate of amoebiasis in Ontario, from 2005 to 2014, to be 5.1 to 6.4 cases per 100,000 populations. In 2014, 741 were confirmed and probable cases of amoebiasis were reported [12]. Furthermore, incidence rates were higher in males 20 years of age or older compared to females of all age groups in 2014. The peak rate occurred in males 40 to 49 years of age (14.7 per 100,000 vs. female rate of 3.3 per 100,000). The most commonly reported risk factor was travel outside the province, and the most frequently reported travel destination included India and Pakistan [12].

In the United States, there is a lack of data regarding amoebiasis-related morbidity and mortality. Gunther and colleagues [13] analyzed death certificate data from 1990 to 2007 to assess the prevalence of amoebiasis-related deaths. A total of 134 deaths were identified, with rates being highest in males, Hispanics, Asian/Pacific islanders, and persons 75 years of age and older. Over 40% of fatal amoebiasis occurred in California and Texas. In 2007, the California Department of Public Health [14] reported 411 cases of amoebiasis in this state alone and estimated the prevalence of* E. histolytica* infection in the United States to be approximately 4%.

### 3.2. Pathogenesis

*Entamoeba histolytica* is an invasive enteric protozoan [1, 2, 10]. Infection typically begins with the ingestion of mature, quadrinucleated cysts found in fecally contaminated food or water. Excystation occurs in the small intestine with the release of motile trophozoites, which migrate to the large intestine. Through binary fission, trophozoites form new cysts, and both stages are shed in feces, but only cysts have the potential to transmit disease due to the protection conferred by their wall [1, 10]. Cysts can survive days to weeks in the external environment, while trophozoites are rapidly destroyed once outside the body or by gastric secretions if ingested [1–3].

Trophozoites have the capacity to adhere and lyse the colonic epithelium and subsequently spread hematologically through the portal vein system to distant sites such as the peritoneum, liver, lung, or brain [7, 11]. Adherence to the colonic mucus layer and colonization is through the Gal/GalNAc lectin, which targets galactose and N-acetyl-D-galactosamine residues found on O-linked sugar side chains of mucins [1, 15]. Mammals that do not carry N-terminal galactose or N-acetyl-D-galactosamine are resistant to trophozoite adherence, providing some degree of immunity against invasive disease [7].

Virulence among* E. histolytica *species is currently under investigation, and the presence of certain enzymes has been linked to increased risk for invasive disease [5, 7, 9, 15, 16]. For instance, glycosidases such as sialidase, N-acetylgalactosamidase, and N-acetylglucosaminidase are needed to remove the branched polysaccharides from mucin cells [15]. This allows the trophozoites to degrade the protective mucous barrier and subsequently penetrate the colonic epithelium increasing the risk of metastasis to distant sites [15]. In a transcriptone analysis study, virulence of* E. histolytica *was also determined by the presence of the enzyme glycoside hydrolase B-amylase. Species lacking this enzyme were unable to breach the mucus barrier and cause invasive disease [15, 16]. Other mechanisms involved in the killing of the epithelial cells and inflammatory cells, including secretion of proteinases (cysteine proteinase), contact-dependent target cells lysis, apoptosis and the formation of amebapores causing cytolysis of infected cells [1, 7, 15, 16].

Several hypotheses have been posited to explain why certain patients have asymptomatic disease, while others progress to invasive disease and are currently being tested. Strain virulence, environment, and the host's genetic susceptibility, immune status, age, and gender have all been found to predict disease severity [1, 5, 9].

Recent studies have shown that the interaction between the host's intestinal flora and* E. histolytica *may mediate pathogenic behavior, generating more virulent strains [17–19]. Galvan-Moroyoqui and colleagues [20] demonstrated that enteropathogenic bacteria can increase Gal/GalNAc lectin expression in* E. histolytica* trophozoites, resulting in increased adhesion capacity and cytopathic effects. Production of proinflammatory cytokines was also augmented in the presence of certain gut bacteria, causing further epithelial damage and facilitating trophozoite invasion [20].

The production of mucosal immunoglobulins (Ig), particularly secretory IgA, plays an important role in the host's gut immune response. Secretory IgA is secreted by plasma cells within the lamina propia and aid in preventing pathogens from adhering and penetrating the mucosal barrier. Significant evidence [1, 21, 22] suggests that mucosal anti-Gal/GalNAc lectin IgA plays a critical role in resistance against amoebic colonization and invasion. In an observational study of children from Bangladesh, Haque and colleagues [22] have shown a correlation between the presence of stool IgA Gal/GalNAc lectin specific antibodies and reduced reinfection rates with* E. histolytica.* Similarly, Abd-Alla and colleagues [23] demonstrated that patients with ALA had greater number and longer duration of anti-Gal/GalNAc lectin IgA, proposing these patients develop an increased and prolonged immune response.

Another recent study conducted by Bernin and colleagues [9] analyzed and compared the sera of patients with ALA, asymptomatic carriers of* E. histolytica* and healthy* E. dispar* infected patients. They found that patients with ALA and asymptomatic carriers both had high titers of IgG and all its subclasses, as well as IgA, suggesting that a strong immune reaction takes place in asymptomatic carriers. Furthermore, sex specific analyses revealed that female asymptomatic carriers had significantly higher titers of IgG and IgG1 subclass in comparison to male asymptomatic carriers [9]. This strong complement-mediated mechanism may be another factor responsible for the decreased prevalence of ALA in the female population [9].

### 3.3. Entamoeba dispar

The* Entamoeba dispar* species is morphologically similar to* E. histolytica,* but due to antigenic and genetic differences, it has been classified as nonpathogenic species that usually infects humans but does not cause disease [17]. Until recently, emerging reports have demonstrated the presence of* E. dispar *in patients with ALA and symptomatic colitis, suggesting that this species may be pathogenic [5, 17]. Using molecular DNA sequencing and genotyping of different* E. dispar* and* E. histolytica* strains, studies have shown the presence of major virulence factors in both species [17–19]. For instance, both species have Gal/GalNAc lectin and cysteine proteases; however, the amount of enzyme produced and secreted, as well as some conformational differences, was found [17, 24]. Various* in vivo* and* in vitro* studies [17–19] also show that cultural and environmental factors play a significant role in the pathogenic behavior of various* E. dispar* strains, as seen in* E. histolytica* strains. Dolabella and colleagues [17] hypothesized that coinfection with both* E. dispar* and* E. histoly*t*ica* may also lead to a recombination event, enhancing its virulence. Given the small number of studies, the pathogenic potential of* E. dispar* still remains controversial. Further research is needed because treatment will be indicated for infected patients if* E. dispar* is indeed shown to cause damage to the human intestine and liver.

### 3.4. Clinical Manifestations

Approximately ninety percent of* Entamoeba* infections are asymptomatic [1, 2, 10]. Risk factors that are associated with increased disease severity and mortality include young age, pregnancy, malignancy, malnutrition, alcoholism, and corticosteroid use [10, 11].

Amoebic colitis generally has a subacute onset, with symptoms that can range from mild diarrhea to severe dysentery, with abdominal pain and watery or bloody diarrhea [1]. Symptoms tend to be nonspecific and the differential diagnosis is broad. Infectious causes that need to be excluded include shigella, salmonella, campylobacter, and enteroinvasive and enterohemorrhagic* Escherichia coli* [1, 25]. Noninfectious causes include inflammatory bowel disease, intestinal tuberculosis, diverticulitis, and ischemic colitis [25].

Unusual but serious complications such as fulminant necrotizing colitis, toxic megacolon, and fistulizing perianal ulcerations can occur, especially when diagnosis and treatment is not timely [1, 26, 27]. Patients that develop necrotizing colitis have a mortality rate of 40% and those with concomitant liver abscess mortality increase to 89% [28, 29]. These patients appear toxic, with fever, bloody diarrhea, and signs of peritoneal irritation [26, 30]. The development of toxic megacolon has been linked to corticosteroid use and is unresponsive to antiamoebic therapy, requiring immediate surgical intervention [30, 31]. Exclusion of inflammatory bowel disease is exceptionally important, given that misdiagnosis and treatment with corticosteroids can lead to these serious complications.

The formation of an ameboma is another uncommon manifestation that may occur in amebic colitis. It tends to present with pain and swelling in the right iliac fossa, or with symptoms of bowel obstruction [27, 32]. Macroscopically, amebomas resemble a mass (or multiple masses) typically localized in the cecum or ascending colon and consist of localized hyperplastic granulation tissue [27, 32]. Ameboma formation has been generally associated with untreated or partially treated amoebic colitis [27]. Given that its appearance can resemble lymphoma, neoplasm, tuberculosis, abscess, or inflammatory bowel disease, colonoscopy and histopathological examination of the biopsied material are warranted to exclude other sinister lesions [27, 30].

Amebic liver abscess is the most common extra intestinal manifestation of amoebiasis [7]. Approximately 50-80% of individuals with ALA will present with symptoms within 2 to 4 weeks, with fever and constant, aching right upper quadrant pain [1, 7, 11]. In up to 50% of cases, patients present more chronically with protracted diarrhea, weight loss, and abdominal pain. Cough, right-sided pleural pain, and subsequent pleural effusion may occur when the diaphragmatic surface of the liver is involved [1]. Dysentery is the most common associated symptom, present in nearly 40% of affected patients [33]. Leukocytosis, transaminitis, and elevated alkaline phosphatase on laboratory evaluation are usually present and imaging reveals an abscess, typically on the right hepatic lobe [7, 11]. Amoebic abscesses tend to be solitary, but multiple abscesses can occur and have been described in previous literature [34, 35]. Anemia and hypoalbuminemia are very common in ALA in comparison to pyogenic abscesses [34].

The lungs are the second most common extra intestinal organ affected [36].

Pulmonary amoebiasis generally occurs by direct extension of an ALA but can also occur by direct hematogenous spread from intestinal lesions or by lymphatic spread [36, 37]. The right lower or middle lobe of the lung is most commonly affected. Patients present with fever, hemoptysis, right upper quadrant pain, and referred pain to the right shoulder or intrascapular region. Pulmonary abscesses, bronchohepatic fistula, and empyema can occur when a liver abscess ruptures into the pleural space [36]. Patients characteristically present with “anchovy sauce-like” like pus or sputum. The presence of bile in these secretions indicates liver origin [36].

Rupture of the liver abscess into the pericardium is also a rare complication with high mortality [38]. It can present acutely with cardiac tamponade resulting from purulent pericarditis, or with a slowly accumulating pericardial effusion. Symptoms include severe chest pain, shortness of breath, and edema from congestive heart failure or constrictive pericarditis [38]. Inferior vena cava (IVC) thrombosis is another extremely rare complication of ALA [29, 36]. Mechanical compression of the IVC by a large hepatic abscess or by erosion from a posterior liver abscess can lead to embolism of the IVC and thromboembolic disease of the lungs [36, 39].

### 3.5. Diagnosis

The World Health Organization (WHO) ranks diarrheal disease as the second most common cause of morbidity and mortality in children in the developing countries [40]. In 1997, the WHO stated the necessity to diagnose and treat all infections with* E. histolytica* and highlighted the demand for new diagnostic techniques for faster and more accurate testing [40]. Various diagnostic tools exist for the diagnosis of* E. histolytica* including microscopy, serology, antigen detection, molecular techniques, and colonoscopy with histological examination. Identification of cysts or trophozoites in stool cannot accurately identify the disease caused by* E. histolytica*, because it is morphologically indistinguishable from* E. dispar* and* E. moshkovskii* which are considered nonpathological species [2].

The identification of* E. histolytica*-specific nucleic acids by PCR is quick, accurate, and effective in diagnosing both intestinal and extra intestinal disease [2]. It has high sensitivity and specificity; however, due to lack of standardization and high cost, it is not yet widely available for diagnostic testing [41]. Stool and serum antigen detection assays are sensitive, specific (differentiating between strains), and easy to preform and can potentially diagnose early infection [42]. These assays use monoclonal antibodies to bind to epitopes found on* E. histolytica* which are not present on other nonpathogenic strains. Antigen detection can be done using ELISA, radioimmunoassay, or immunofluorescence [42].

The most sensitive serology assay is the indirect hemagglutination test (IHA) and is positive in up to 90% of patients with intestinal disease [43]. Unlike* E. dispar*, infection by* E. histolytica* results in the development of antibodies which can be detected within 5 to 7 days from acute infection [43]. However, up to 35% of individuals from endemic areas have persistent antibodies from previous infection; therefore, only a negative serology result can be helpful to exclude disease [26, 43].

Finally, direct visualization of the colon by colonoscopy can be performed to diagnose amoebiasis, particularly when nonspecific gastrointestinal symptoms make diagnosis difficult [44]. It is also useful in excluding other disease, particularly neoplasms. The diagnostic value of the colonoscopy lies in the ability to take biopsies and microscopically identify intestinal amoebiasis. Lee and colleagues [44] found right-sided colitis and proctosigmoiditis varied in colonoscopic appearance. Right-sided disease included erosions, ulcers, exudates, or edematous cecal mucosa, while findings for proctosigmoiditis were swollen, edematous mucosa with bloody exudate [44]. The most common findings are “flask-like” ulcerations or erosions typically present in the cecum, followed by the rectum, ascending colon, sigmoid colon and, rarely, the transverse and descending colon [44, 45].

Intestinal tuberculosis and inflammatory bowel disease can present similar to amoebic colitis. Endoscopic features such as cryptitis and crypt abscesses, as well as erosions and ulceration of the rectum, which are commonly seen in ulcerative colitis, may also be present in amebic colitis [25, 44]. The intervening mucosa between the amebic ulcers may appear normal, therefore mimicking Crohn's disease and intestinal tuberculosis [25]. Because the cecum is the most commonly affected area in amoebiasis, a normal terminal ileum, transverse and descending colon can help distinguish it from IBD and infectious colitis [44]. In addition, enemas given prior to colonoscopy may wash away the exudate containing the amoeba or lyse the trophozoites which may lead to failure of identification of the organism on biopsy [25].

Combining serological testing with PCR or antigen detection is currently the best diagnostic approach [2]. Using combined technique will increase the specificity and sensitivity in the diagnosis of* E. histolytica *infection. Further, this method allows clinicians to distinguish acute infection from chronic or previously treated infection.

### 3.6. Treatment

Specific diagnosis and treatment is warranted in all infections caused by* E. histolytica*, even in asymptomatic carriers, not only because of the potential of developing invasive disease, but also to diminish the spread of disease [1]. Noninvasive colitis may be treated with only a luminal agent such as paromycin, to eliminate intraluminal cysts [1]. For invasive amoebiasis and extra intestinal disease, nitroimidazoles (e.g., metronidazole) are the mainstay therapy but are only active against the trophozoite stage [1, 26]. Nitroimidazoles with longer half-lives, such as tinidazole, secnidazole, and ornidazole, allow for shorter treatment periods and tend to be better tolerated but are not available in the United States. After a 10-day course of a nitroimidazole, paromycin should be given to assure that the luminal parasites are cleared to prevent relapse [26]. It has been shown that 40-60% of patients have persistent intestinal parasites after treatment with only nitroimidazole. Second line luminal agents include diiodohydroxyquin and diloxanide furoate [1, 26].

If fulminant amoebic colitis develops, broad-spectrum antibiotics should be added to the treatment due to risk of bacterial translocation [26]. Surgical intervention is rarely necessary and reserved for those patients with signs of acute abdomen or those with toxic megacolon. In certain instances, aspiration of ALA is required, particularly when there is no clinical response after five to seven days of antiamoebic therapy. Patients with high risk of abscess rupture (cavity diameter of ≥ 5 cm and left lobe abscesses) or large amoebic pleural effusions should also be considered for drainage. Imaging-guided percutaneous needle aspiration or catheter drainage is the procedure of choice [36].

Education regarding the importance of hand washing and hygiene is the single most important measure in preventing the spread of amoebiasis, as well as other infectious diseases. It is estimated that washing hands with soap and water could reduce diarrheal disease-associated mortality by up to 50%, particularly after using the toilet, changing diapers, and before handling or preparing food (3). However, practicing personal hygiene may be difficult in many areas of the world due to lack of resources such as clean water and soap.

### 3.7. Vaccine

Vaccines are currently being investigated in rodent and nonhuman primate models and appear promising. Using both native and recombinant forms of the amoebic Gal/GalNAc lectin has demonstrated protection against intestinal and liver infection [21]. Alla and colleagues [45] investigated a vaccine in baboon models, a natural host for* E. histolytica*. Cholera toxin B subunit was coadministered with Gal-lectin which resulted in significant, moderate level of protection against* E. histolytica* reinfection. Vaccinated baboons displayed higher titers of intestinal antipeptide IgA, intestinal antilectin IgA, and serum antipeptide IgG antibodies, and follow up colonoscopy showed no sign of inflammatory colitis nor amoebic invasion [45]. Targeting other components of* E. histolytica, *such as serine-rich protein and the 29-kDa-reductase antigen, has been shown successful against ALA in rodent models [21].

More rigorous testing for the development of a successful vaccine against* E. histolytica *is warranted. Aside from identifying target immunogenic proteins, the combination of doses, adjuvants, and boosts needs to be optimized [26]. The responses in animal models, nonhuman primates in particular [45], suggest that it may be possible to develop a viable human vaccine, advancing to get beyond the preclinical stage. Unfortunately, however, the induction of long-term memory has not yet been demonstrated in animal models, which is the hallmark of a successful vaccine.

## 4. Conclusion

Amoebiasis continues to be a large health issue in developing countries, particularly in children. With increased travel and emigration to developed countries from endemic areas, the incidence and prevalence of amoebiasis continue to increase. Since most patients are asymptomatic, diagnosis and treatment can be challenging for clinicians, potentially leading to continuous spread of the disease.* E. histolytica* should be considered as a differential diagnosis of colitis and with certain extra intestinal manifestations, particularly when certain demographics (i.e., gender, race, travel history) are present.

Surprisingly little is still known about the immune response underlying invasive amoebiasis, as well as the factors that allow certain people to remain as asymptomatic carriers while others progress to disseminated disease. As more evidence is gathered to elucidate the pathogenesis of* E. histolytica,* and the immune response mounted against this infection, more potential target proteins can be investigated in the development of a vaccine. The fact that certain people are able to mount partial immunity against intestinal infection remaining asymptomatic indicates that the potential of developing a successful vaccine is promising.

Studies are investigating various immunoglobulins, cytokine and chemokine titers among asymptomatic carriers and those with invasive disease [9, 21, 22]. As mentioned previously, strong complement-mediated mechanisms may be the reason females tend to be more resistant in developing ALA. Therefore, using the combinations of target proteins (e.g., Gal-lectin, which prevents adhesion and invasion of the amoeba), and adjunct proteins that harness both humoral and cell mediated immune response, may facilitate the development of a vaccine. Further research is needed to evaluate the efficacy of immune response and protection in animal models at later time points, in order to assess immunological memory. Finally, the assumption that* E. histolytica *is the only pathogenic species capable of causing disease should be reconsidered. New evidence demonstrates that* E. dispar* is a potential pathogen capable of causing disease [17–19]. Therefore, new treatment guidelines may be warranted.
